# Whole-genome assembly and analysis of a medicinal fungus: *Inonotus hispidus*

**DOI:** 10.3389/fmicb.2022.967135

**Published:** 2022-09-06

**Authors:** Shaojun Tang, Lei Jin, Pin Lei, Chenxia Shao, Shenlian Wu, Yi Yang, Yuelin He, Rui Ren, Jun Xu

**Affiliations:** Hunan Institute of Microbiology, Changsha, China

**Keywords:** *Inonotus hispidus*, functional annotation, CAZyme, secondary metabolites, phylogenetic analysis

## Abstract

*Inonotus hispidus* (*I. hispidus*) is a medicinal macrofungus that plays a key role in anti-tumor and antioxidant functions. To further understand and enhance the value of *I. hispidus*, we performed whole-genome sequencing and an analysis of its strain for the first time. *I. hispidus* was sequenced using the Illumina NovaSeq high-throughput sequencing platform. The genome length was 35,688,031 bp and 30 contigs, with an average length of 1,189,601.03 bp. Moreover, database alignment annotated 402 CAZyme genes and 93 functional genes involved in regulating secondary metabolites in the *I. hispidus* genome to find the greatest number of genes involved in terpenes in that genome, thus providing a theoretical basis for its medicinal value. Finally, the phylogenetic analysis and comparative genomic analysis of single-copy orthologous protein genes from other fungi in the same family were conducted; it was found that *I. hispidus* and *Sanghuangporus baumii* have high homology. Our results can be used to screen candidate genes for the nutritional utilization of *I. hispidus* and the development of high-yielding and high-quality *I. hispidus* via genetic means.

## Introduction

*Inonotus hispidus* (*I. hispidus*) is known as Shaggy bracket or Shaggy polypore due to the hairy shape of its fruiting bodies (Song et al., 2021). *I. hispidus* is a valuable medicinal fungus that mainly grows on mulberry, ash, elm, poplar, and Japanese locust (Liu et al., 2019b); it is found in Hunan, Heilongjiang, Jilin, Liaoning, Inner Mongolia, and Xinjiang in China. Exploration of its chemical composition has shown that *I. hispidus* is rich in polysaccharides, polyphenols, steroids, terpenes, fatty acids, amino acids, and other active ingredients, and it is rich in pigment substances (Politi et al., 2007). Because of the richness of these active ingredients, *I. hispidus* plays an essential role in medicine (Benarous et al., 2021; Zhang et al., 2021). In Chinese history, it is recorded that *I. hispidus*, as a traditional Chinese medicine, has anti-inflammatory, hypoglycemic, hypolipidemic, and blood-activating and stasis-removing effects (Gründemann et al., 2016). This coincides with the research results of modern medicine, and many medical studies have explored the pharmacological mechanism of *I. hispidus* (Awadh Ali et al., 2003; Benarous et al., 2015). Gründemann et al. explored the effect of *I. hispidus* extract on different types of human immune cells using flow cytometry; the results showed that the extract exerted an immunomodulatory influence (Gründemann et al., 2016). Another study found that (4*S*,5*S*)-4-Hydroxy-3,5-dimethoxycyclohex-2-enone (HDE), isolated and purified from *I. hispidus*, can inhibit proliferation of HepG2 cells and has an anti-tumor effect (Yang et al., 2019). In addition to targeting liver cancer, *I. hispidus* extracts have inhibited the proliferation of various tumor cells and has significantly improved the immune function of the host organism (Wang et al., 2016; Li and Bao, 2022). The polysaccharides and polyphenols in *I. hispidus* exhibit excellent antioxidant and anti-inflammatory effects and can strongly scavenge DPPH and OH free radicals (Zan et al., 2011; Liu et al., 2019a).

At present, most of the related research on *I. hispidus* focuses on the extraction of its active ingredients or its functional roles, and there is very little content about its genome sequencing. In recent years, more studies have reported on the results of fungal genome sequencing. Genome-wide sequencing of fungi from Hymenochaetaceae has revealed their biological activity value (Huo et al., 2020; Jiang et al., 2021). The genome sequencing results also provide a strong research basis for our understanding of fungal function and ingenuity. Through the sequencing analysis of the genome library, we can learn about the gene regulatory network, biosynthetic pathway, and drug characteristics of these large fungi, which creates a good foundation for their subsequent commercial production and the enrichment of their medical value. In the study discussed in this paper, we developed a genome-wide map of *I. hispidus* (CGMCC 21046). The genes of *I. hispidus* were annotated through public and proprietary databases to identify the fungus’ potential medicinal functional roles. Then, *I. hispidus* was compared with the genome information of *Pyrrhoderma noxium* (*P. noxium*), *Fomitiporia mediterranea* (*F. mediterranea*), *Sanghuangporus baumii* (*S. baumii*), *Phellopilus nigrolimitatus* (*P. nigrolimitatus*), *Phellinidium pouzarii* (*P. pouzarii*), *Schizopora paradoxa* (*S. paradoxa*), *Rickenella mellea* (*R. mellea*), *Wolfiporia cocos* (*W. cocos*), *Ganoderma sinense* (*G. sinense*), *Polyporus arcularius* (*P. arcularius*), *Fomes fomentarius* (*F. fomentarius*), *Trametes cingulate* (*T. cingulate*), *Trametes coccinea* (*T. coccinea*) and *Trametes cinnabarina* (*T. cinnabarina*) belonging to the Hymenochaetales order of fungi to further reveal the evolution and species dynamics of *I. hispidus*.

## Materials and methods

### Fungal material, DNA extraction, and library construction sequencing

**FIGURE 1:**
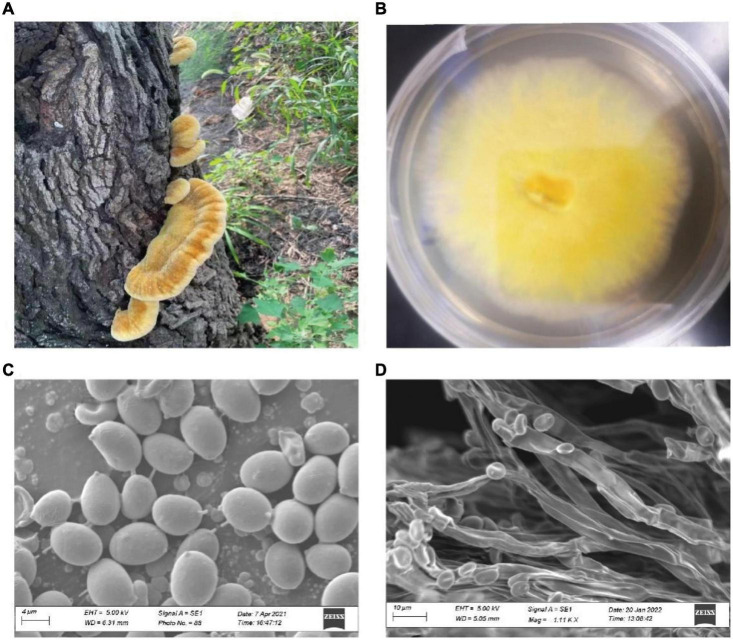
The fruiting bodies of *I. hispidus* and the monokaryotic strain used for genome sequencing. **(A)** The fruiting bodies of *I. hispidus*. **(B)** Mycelia of *I. hispidus.* **(C)** Scanning electron microscope image of *I. hispidus* spores. **(D)** Scanning electron microscope image of *I. hispidus* mycelia.

The fruiting bodies of *I. hispidus* were collected from mulberry trees in Linqing, Shandong Province, China. Specimen was identified by morphologic and molecular analyses (Figure 1; Schwarzer et al., 2003). The specimen of *I. hispidus* was deposited in the China General Microorganism Culture Collection and Management Center (CGMCC 21046). The monokaryotic strain was germinated from one of the spores of the specimen and used for whole-genome sequencing. The mycelia of *I. hispidus* were cultured on an improved potato dextrose agar medium containing 5% wheat bran for ten days at 24°C in the dark and collected for genome sequencing (Tian et al., 2021).

First, the sample was removed from liquid nitrogen, then 1 mL of B1 lysis buffer was added, and then vortexed and mixed. Next, 2 μL of RNaseA, 40 μL of lysozyme, and 45 μL of Proteinase K were added in sequence and mixed in a water bath at 37°C for 60 min. Then, 0.35 mL of B2 lysate was added and mixed in a water bath at 50°C for 60 min. After centrifugation at 12,000 rpm for 5 min, the supernatant was stored for later use.

Next, 2 mL of QBT (QBT: 43.83 g NaCl and 10.46 g MOPS (acid-free) were dissolved in 800 mL water, then the solution pH was adjusted to 7.0, 150 mL pure isopropanol and 15 mL 10 % Triton-100 were added to the final volume to 1 L.) was added to the equilibrated 20 G column to allow it to flow out by gravity; treated lysate was added to the column to allow it to flow out by gravity, then 1 mL of QC (QC: 58.44 g NaCl and 10.46 g MOPS were dissolved in 800 mL water, then pH was adjusted to 7.0, 150 mL pure isopropanol was added to the final volume to 1 L.) was added to the cleaning column to allow it to flow out by gravity for a total of three washes; 1 mL of QF (QF: 73.05 g NaCl and 6.06 g Tris-base are dissolved in 800 mL water, then the pH value of the solution is adjusted to 8.5, 150 mL pure isopropanol is added to the final volume to 1 L) was added and used to elute the DNA; 0.7-times the volume of isopropanol was added to precipitate the DNA; the sample was centrifuged at 12,000 rpm for 20 min at 4°C, then the supernatant was discarded, the DNA pellet was collected, and 1 mL of 70% ethanol was added to wash the pellet. The pellet was washed twice; an appropriate volume of TE (10 mmol/l Tris-HCl pH 8.0, 0.1 mmol/l EDTA) was added to dissolve the DNA, and it was incubated in a metal bath at 37°C for 60–120 min.

The following steps were used to extract high-quality DNA. After the samples passed the quality inspection, the genomic DNA was randomly interrupted; the large fragments of DNA were enriched and purified by magnetic beads, and the large fragments were recovered using cutting gel. End repair and 3′ end adding A was done the reaction product was purified; the fragment repair product was obtained and purified using sequencing-related adapters to obtain the final library. Qubit was used to accurately and quantitatively identify the constructed DNA library. A specific amount of the DNA from the library was mixed with the relevant reagents on the machine and added to the flow cell. Real-time single-molecule sequencing was performed on the PromethION sequencer to obtain the original sequencing data.

### Sequencing data quality control and genome assembly

The raw data format of Nanopore PromethION sequencing data is a binary fast5 format containing all the raw sequencing signals. After base calling with Guppy v. 3.2.6 software, the fast5 format data were converted to the fastq format. After further filtering of the adapters, the low-quality data, and the short fragments (length < 2,000 bp), the total dataset was obtained.

The proportion of clean reads aligned to the reference genome accounts for the total number of clean reads. If the reference genome is properly selected and there is no contamination in the relevant experimental process, the alignment rate of the sequencing reads will be greater than 80%. Moreover, the alignment rate is related to the relative relationship of the reference genome, the quality of the reference genome assembly, and the quality of the reads sequencing. The more reads in the genome, the higher the alignment rate. The comparison uses Burrows-Wheeler Aligner (BWA) v. 0.7.10 software (Li and Durbin, 2009). We used BUSCO v. 2.0 software to assess the integrity of the fungal genome assembly (Simão et al., 2015).

We used four software (LTR_FINDER v. 1.05, MITE-Hunte v. 1.0.0r, RepeatScout v. 1.0.5, PILER-DF v. 2.4) to build a database of repeat sequences in *I. hispidus* genome based on the principle of structure prediction and ab-initio prediction (Edgar and Myers, 2005; Price et al., 2005; Xu and Wang, 2007; Han and Wessler, 2010). The repeat sequence database of the fungal genome was classified by PASTEClassifier v. 1.0, and then merged with the Repbase (19.06) database as the final repeat sequence database (Jurka et al., 2005; Wicker et al., 2007). Next, RepeatMasker v. 4.0.6 software was used and based on the constructed repeat sequence database, the repeat sequence of the fungus was predicted (Tarailo-Graovac and Chen, 2009).

The repetitive sequence is described in the general feature format (GFF), including the source of the repetitive sequence elements (mainly transposons), the locus in the genome, as well as the type, characteristics, and other information. For details, please refer to the description of GFF format at: http://www.sequenceontology.org/gff3.shtml.

Gene structure prediction mainly adopts *de novo* prediction, based on homologous protein and transcriptome evidence, and then integrates the three prediction results. Genscan v. 1.0 (Burge and Karlin, 1997), Augustus v. 2.4 (Stanke and Waack, 2003), GlimmerHMM v. 3.0.4 (Majoros et al., 2004), GeneID v. 1.4 (Blanco et al., 2007), SNAP (version 2006-07-28) (Korf, 2004) were used for *de novo* prediction; homologous protein-based prediction was done using GeMoMa v. 1.3.1. The reference transcript-based assembly was determined using Hisat2 v. 2.0.4 and Stringtie v. 1.2.3, and PASA v. 2. TransDecoder v. 2.0.0.2 was used to predict the UniGene sequence based on the transcriptome assembly; finally, EVM v. 1.1.1 was used to integrate the prediction results obtained by the three methods previously mentioned, and the results were modified with PASA v. 2.0.2. The number and average length of the protein-coding genes was determined, as were the number and average length of the introns and exons. Non-coding RNAs are RNAs that do not encode proteins, including RNAs with known functions, such as microRNA, rRNA, and tRNA. Depending on the structural characteristics of non-coding RNAs, different strategies are used to predict different non-coding RNAs. tRNA in the genome was predicted using tRNAscan-SE v. 1.3.1 software. The rRNA in the genome and ncRNAs other than tRNA and rRNA were predicted using Infernal v. 1.1 software based on the Rfam v. 12.0 database (Nawrocki and Eddy, 2013).

### Genome functional annotation

The predicted gene sequences were compared with the functional databases, such as Clusters of Orthologous Genes (COG) (Tatusov et al., 2000), Gene Ontology (GO) (Ashburner et al., 2000), Kyoto Encyclopedia of Genes and Genomes (KEGG) (Kanehisa et al., 2004), protein families (Pfam) (Finn et al., 2016), and Nr (Deng et al., 2006) for BLAST v. 2.2.29 to obtain the gene function annotation results. Gene function annotation mainly includes a sequence similarity search and a motif similarity search. For the sequence similarity search, diamond BlastP (version: 2.9.0) was carried out between the protein sequence encoded by the full-length gene and the existing protein database Uniprot, RefSeq, and NR. The metabolic pathway was determined using the KEGG database. The parameters (-evalue 1e-5) were compared to obtain functional information about the sequence and the metabolic pathway information that may be involved in the protein. KEGG annotation uses KOBAS (version: 3.0) to associate with KEGG orthology and pathway. The Uniprot database records the correspondence between each protein family and the functional nodes in GO, and it predicts the biological function of the protein sequence encoded by the gene. Based on the association between the databases (Uniprot/Swiss-Port), the annotation information of the eggNOG database is obtained, and the COG annotation results are selected for COG classification statistics and mapping. For the motif similarity search, proteins are generally composed of one or more functional regions, which are usually called domains. Different combinations of domains produce a variety of proteins. Therefore, the identification of protein domains is important for the analysis of protein functions. This was done using a hmmScan (version: 3.1; parameter: e-value).

### Carbohydrate enzyme annotation

Carbohydrates play an important role in many biological functions. A significant amount of meaningful biological information can be obtained by studying carbohydrate-related enzymes. CAZy (a database of Carbohydrate-Active enZYmes [CAZymes]) data focuses on analyzing the genome, structure, and biochemical information of carbohydrate enzymes. HMMER (version: 3.2.1, filter parameter e-value 0.35) was used to annotate the protein sequences based on the CAZy database (Cantarel et al., 2009).

### Cytochrome P450 annotation

Cytochrome P450 (CYP450) is a family of proteins supplemented by heme. They can catalyze the oxidation of many substrates. Due to the maximum absorption wavelength at 450 nm after the reduction state of the protein combined with CO, it is also known as P450. It participates in the metabolism of endogenous substances and exogenous substances, including drugs and environmental compounds. Diamond BlastP (version >: 2.9.0; parameter: –evalue 1e-5) was used to annotate the target protein sequence based on the Fungal Cytochrome P450 Database (FCPD) (Park et al., 2008).

### Transporter Classification Database annotation

Transporter Classification Database (TCDB) is used to classify membrane transport proteins. It develops a transporter classification (TC) system, which is similar to the Enzyme Commission (EC) system for enzyme classification; however, the TC system provides both functional and evolutionary information. TCDB provides a TC number for each transporter family. TC numbers consist of five digits or letters separated by decimals. SignalP (version: 5.0) software was used to analyze the protein sequences of all the predicted genes, and the proteins containing signal peptides were found. TMHMM (version: 5.0) software was used to analyze the protein sequences of all the predicted genes to identify the proteins that contain transmembrane helixes, namely transmembrane proteins. The proteins containing a transmembrane helix were removed from the predicted proteins containing a signal peptide. The remaining proteins are secreted proteins (Saier et al., 2006).

### Prediction of gene clusters involving secondary metabolites

antiSMASH 3.0^1^ was used to analyze the secondary metabolite biosynthetic gene clusters in the fungal genome. Thus, the query and prediction of natural product synthetic gene clusters between genomes can be realized. The parameter settings remain the default parameter values. To validate the predicted results, we manually checked the resulting gene clusters. The genome sequence of the secondary metabolite biosynthesis gene cluster of *I. hispidus* was predicted using the antiSMASH 3.0 database and it was compared with 12 other fungi under the phylum Hymenochaetales (Medema et al., 2011).

### Construction of a fungal genome evolution tree by orthologous single-copy genes and collinearity analysis

The protein sequences of all the genomes were predicted using Prodigal v. 2.6.3 software. Orthofinder v. 2.5.2 software was used to calculate the orthologous single-copy genes according to the above protein sequences. MAFFT v. 7.480 software was used for multiple sequence alignment of the direct homologous single-copy genes (Tian et al., 2021). Finally, Python v. 3.7. script is used to concatenate the aligned protein sequences belonging to each bacterium, and IQ-TREE v. 2.1.3 software was used to calculate the evolutionary tree with the fasta file concatenated mentioned above. The algorithm that was used is the maximum likelihood method. Collinearity analysis was performed using TBTools software, based on location information from the GFF3 files of *I. hispidus, S. baumii, P. nigrolimitatus* and *P. pouzarii*.

### Identification of the homologous genes and cluster analysis of the gene family

Based on all the amino acid sequences of the selected species, OrthoFinder software (version: 2.3.12; parameter: –Mesa) (Emms and Kelly, 2019) was used for gene family clustering, and BlastP software (version: 2.6.0; parameters: –evalue 1e-5 – outfmt 6) (Camacho et al., 2009) was used for the genome alignment. Finally, the gene family identification results were statistically analyzed. Perl script was used to count and map the Wayne diagram based on the clustering results of the protein sequences of the four selected species. The gene family common to all species is called a shared gene. Based on the shared gene and functional annotation (GO and KEGG), R package clusterProfiler was used for the GO and KEGG enrichment analysis. Among all the selected species, the specific gene family is called the unique gene family. Based on the unique gene and functional annotation (GO and KEGG), R package clusterProfiler was used for GO and KEGG enrichment analysis.

## Results

### Sequencing and assembly data

**FIGURE 2:**
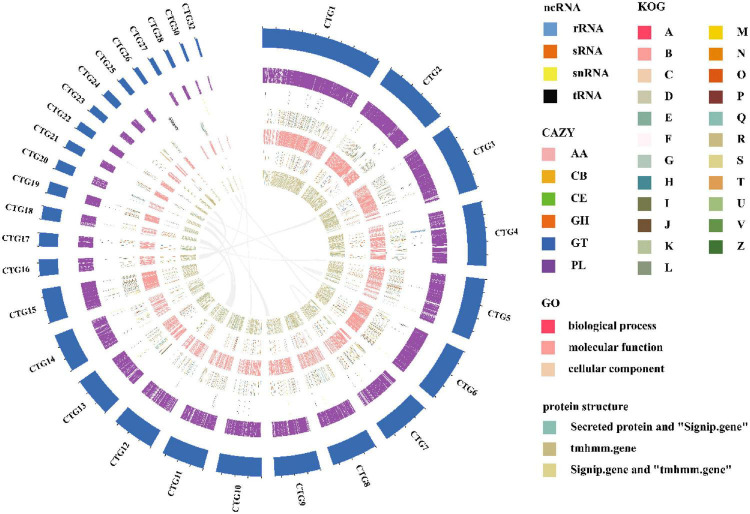
A circular genome map of *I. hispidus*. From outside to the center: circle 1: contig keyboard; circle 2: gene density; circle 3: ncRNA; circles 4–6: the predicted protein-coding genes using KOG, GO, and CAZy databases, respectively, where different colors represent different function classifications; 7th circle: large fragment duplication.

**FIGURE 3:**
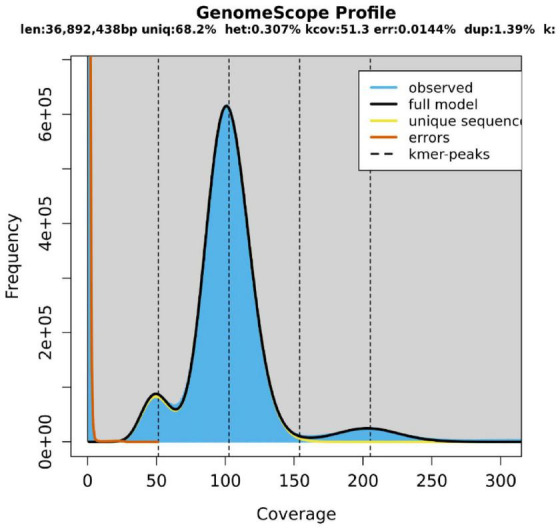
K-mer-Depth and K-mer Species—Frequency Distribution Plot.

**FIGURE 4:**
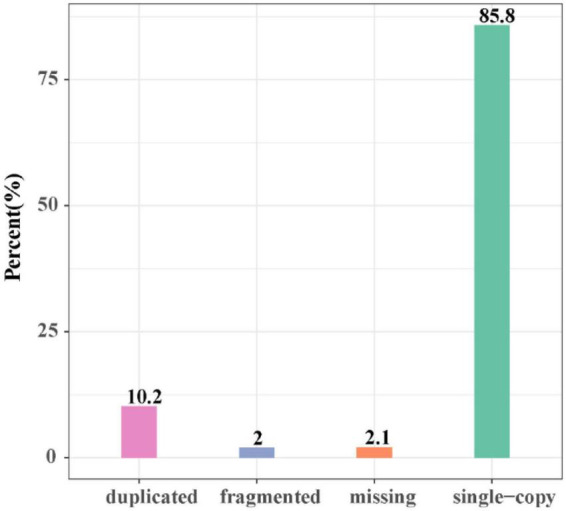
The map of the gene coding gene BUSCO evaluation results.

The length of the genome obtained from Mushroom is 35,688,031 bp, which contains 30 contigs; the average contig length is 1,189,601.03 bp, the longest contig is 4,604,913 bp, and the guanine-cytosine (GC) content is 48.53%. The specific data information of sequencing is shown in Table 1. The sequenced reads were analyzed using K-mer methods to estimate genome size and heterozygosity. The genome assembly results are shown in Table 2 and Figure 2. The map rate, average depth, and coverage in the sequencing are 99.33%, 152.52 and 99.75%, respectively. The K-mer results are shown in Figure 3. Finally, we used BUSCO software (version: 4.1.4) to assess the completeness of the genome assemblies based on the fungi database (fungi_odb10). The completeness of the genome assembly and the annotation of single-copy ortholog test results indicated that the annotation set was very complete, with 85.8% of the fungal BUSCO present in the RefSeq annotation set and 2% of the genes were fragmented (Figure 4).

**TABLE 1:** Statistics of the data volume of the gene sequencing.

Rank	Flag	Total base	Total reads	Max length	Average length	N50	L50	N90	L90	mean Q
>0	all	6,589,417,267	774,294	352,372	8,510.22	21,509	86,223	3,198	415,299	10.87
>0	pass	6,118,865,277	713,582	157,122	8,574.85	21,682	79,604	3,217	382,819	11.31
>0	fail	470,551,990	60,712	352,372	7,750.55	19,231	6,666	2,977	32,541	5.77
>5,000	all	5,460,879,454	297,703	352,372	18,343.38	27,327	62,960	7,889	210,289	10.78
>5,000	pass	5,078,080,879	275,712	157,122	18,418.06	27,490	58,298	7,910	194,541	11.17
>5,000	fail	382,798,575	21,991	352,372	17,407.05	25,338	4,684	7,639	15,756	5.83
>10,000	all	4,601,005,374	174,948	352,372	26,299.27	32,095	48,444	13,628	135,517	10.71
>10,000	pass	4,285,318,818	162,493	157,122	26,372.32	32,231	44,980	13,655	125,807	11.09
>10,000	fail	315,686,556	12,455	352,372	25,346.17	30,436	3,474	13,299	9,714	5.83
>50,000	all	1,021,705,777	16,272	352,372	62,789.19	61,352	6,993	51,904	14,267	10.67
>50,000	pass	960,086,542	15,293	157,122	62,779.47	61,369	6,573	51,904	13,409	10.97
>50,000	fail	61,619,235	979	352,372	62,940.99	61,101	420	51,899	859	5.87
>100,000	all	23,257,716	206	352,372	112,901.53	108,725	95	101,071	183	9.93
>100,000	pass	21,050,260	189	157,122	111,377.03	108,563	88	101,000	169	10.33
>100,000	fail	2,207,456	17	352,372	129,850.35	117,568	7	104,455	15	5.46

**TABLE 2:** Statistics of the assembly results.

Item	Value
Total length (bp)	35,688,031
Total length without N (bp)	35,688,031
Total number	30
GC content (%)	48.53
N50 (bp)	1,831,239
N90 (bp)	514,575
Average (bp)	1,189,601.03
Median (bp)	960,179.50
Min (bp)	69,266
Max (bp)	4,604,913

### Gene annotation

Gene prediction mainly uses BRAKER software (version: 2.1.4). First, Gene Mark-EX was used to train the model, and then AUGUSTUS was used for the prediction. The protein coding region contains a total of 12,671 genes, and the average length of the mRNA is 1,796.85 bp. The average number of exons per gene was 6.59, and the average lengths of exons and introns were 213.25 and 70.03 bp, respectively. For the non-coding RNAs, we predicted 17 rRNAs, 1 sRNA, 16 snRNAs, and 88 tRNAs, where the average length of r RNA is 1,836 bp.

**FIGURE 5:**
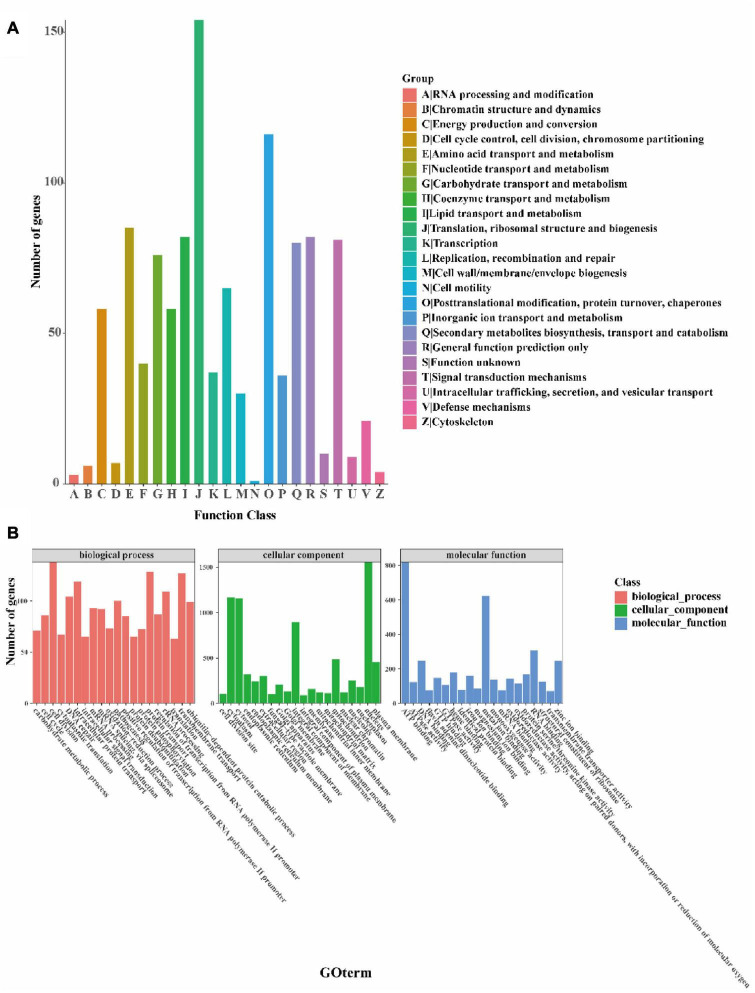
Functional annotation of the *I. hispidus* genome. **(A)** COG feature annotation classification chart. **(B)** GO functional annotation classification statistics plot.

The orthology of the *I. hispidus* genes was classified using the COG database. We found that the top five enriched categories were: “translation, ribosomal structure and biogenesis,” “posttranslational modification, protein turnover, chaperones,” “amino acid transport and metabolism,” “lipid transport and metabolism,” and “general function prediction only SIFunction unknown” (Figure 5A). Next, we simplified the GO annotation information to obtain the GOslim classification. After summarizing the functions of the genes from the three aspects of biological processes, cellular components, and molecular functions, we selected the top 20 annotations under each classification. The most abundant secondary classification of GOslim was drawn, and it was found that the “cell division” has the highest gene enrichment in the biological process, the “nucleus” has the highest gene enrichment in the cellular component, and the “ATP binding” has the highest gene enrichment in molecular (Figure 5B).

**FIGURE 6:**
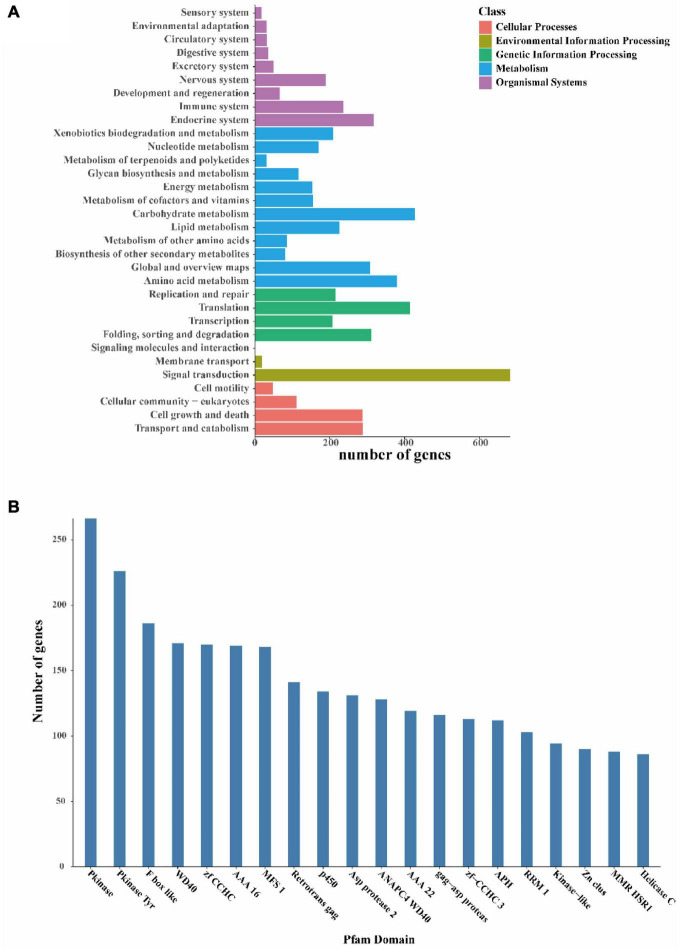
Functional annotation of the *I. hispidus* genome. **(A)** KEGG Pathway functional classification diagram. **(B)** Pfam functional annotation classification statistics plot.

Finally, to further understand the gene functions in *I. hispidus*, we annotated the sequences with KEGG and classified them according to the KEGG metabolic pathways in which they were involved. We found that the gene function with the highest enrichment in the organic systems classification is the “endocrine system,” the gene function with the highest enrichment in the metabolism classification is “carbohydrate metabolism,” and the gene function with the highest enrichment in the genetic information processing classification is “translation.” The most enriched gene function in the Environmental Information Processing classification is “signal transduction,” and the most enriched gene function in the Cellular Processes classification is “transport and catabolism” (Figure 6A). We also identified 266 Pkinases, 226 Pkinase Tyr, 186 F-box-like, etc., in the Pfam domain of the *I. hispidus* genome (Figure 6B).

### Carbohydrate-active enzymes

**FIGURE 7:**
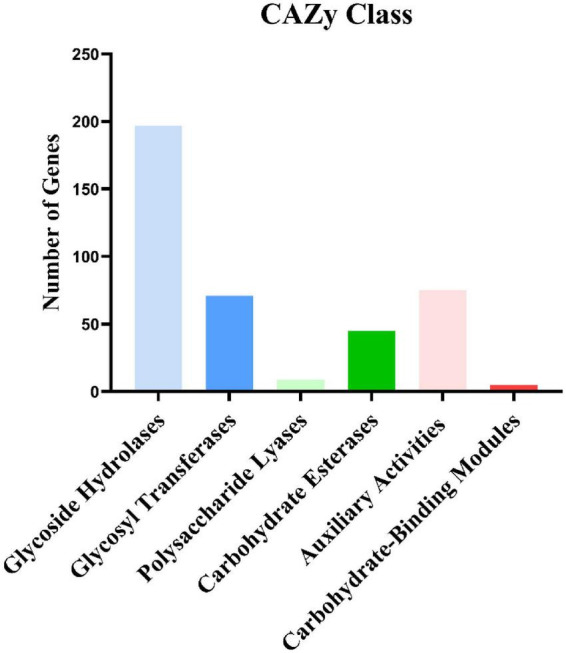
Carbohydrate activity enzyme annotation results.

We annotated the *I. hispidus* protein sequences based on the CAZyme database with HMMER (version: 3.2.1, filter parameters E-value < 1e-18; coverage > 0.35). The results showed that the genes were significantly enriched in the glycoside hydrolase family. A total of 402 CAZyme-encoding genes were annotated, including 121 superfamilies. Among the 402 CAZyme-encoding genes, 197 belong to glycoside hydrolases, 71 belong to glycosyl transferases, nine belong to polysaccharide lyases, 45 belong to carbohydrate esterase, 75 belong to auxiliary activities, and five belong to carbohydrate-binding modules (Figure 7).

### Protein structure prediction

We obtained 999 protein sequences by cytochrome P450 annotation. We obtained 1,408 membrane transport-related proteins through TCBD annotation and found 733 proteins containing signal peptides. We identified 2,305 transmembrane proteins and 495 secreted proteins (Table 3).

**TABLE 3:** Statistical table of protein structure prediction.

Protein type	Number
CYP450	999
TCDB	1408
Signal peptide protein	733
Transmembrane protein	2305
Secreted protein	495

### Secondary metabolites

**FIGURE 8:**
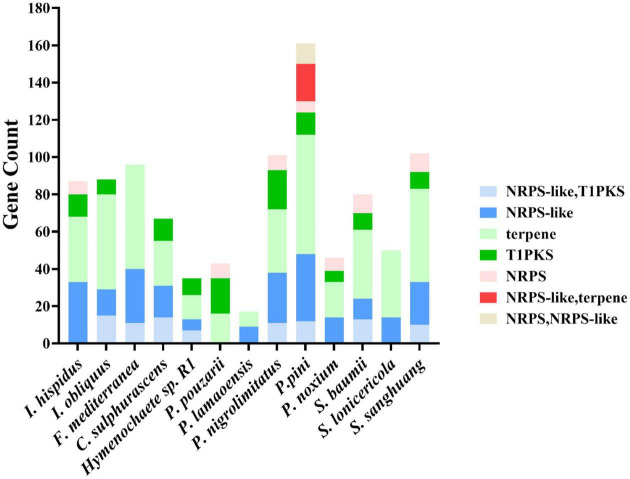
Genome sequence comparison of the secondary metabolite biosynthetic gene clusters.

Prediction of the genome sequence of the secondary metabolite biosynthesis gene cluster using the AntiSMASH database found that 87 functional genes are involved in regulating the production of secondary metabolites. Among them, 33 functional genes regulated the production of NRPS-like, 35 functional genes regulated the production of terpenes, 12 functional genes regulated the production of T1PKS, and seven functional genes regulated NRPS. It was found that the other fungal genomes of Hymenochaetaceae mainly regulated the production of NRPS-like, terpene, T1PKS, and NRPS. However, some genes in other species could simultaneously regulate NRPS-like, T1PKS, or NRPS-like, terpene or NRPS, NRPS-like (Figure 8).

### Comparative genomic analysis of *Inonotus hispidus*

**FIGURE 9:**
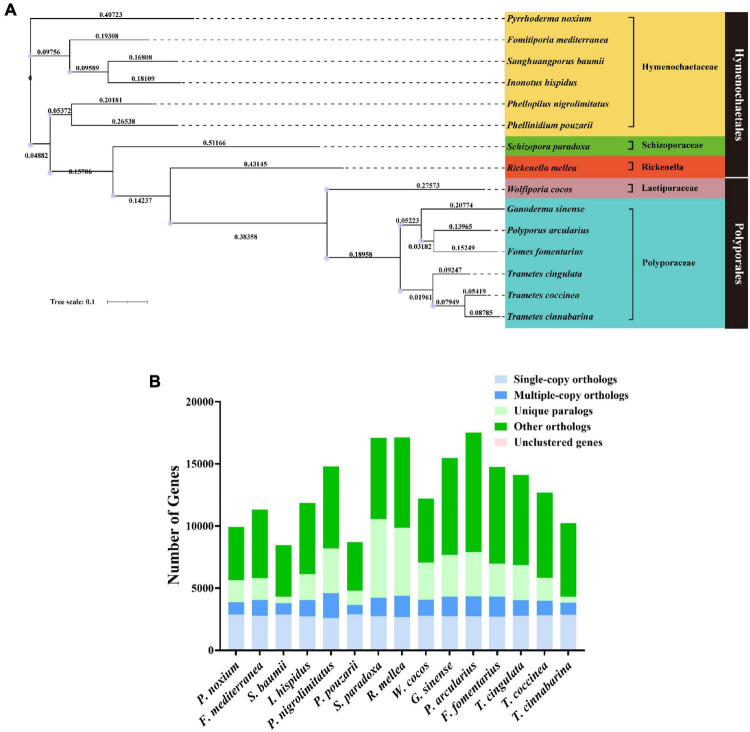
Comparative genomic analysis of *I. hispidus* and other 14 fungi. **(A)** Phylogenetic tree of *I. hispidus* and 14 other fungal species. **(B)** Homologous genes of *I. hispidus* and 14 other fungi species.

**FIGURE 10:**
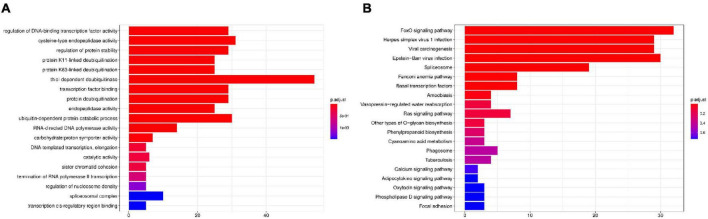
Gene family enrichment analysis of *I. hispidus* compared with other 14 fungi. **(A)** GO enrichment results of the share gene family. **(B)** KEGG enrichment results of the shared gene family.

We constructed a phylogenetic tree using single-copy orthologous protein genes from *I. hispidus* and 14 other fungal species. The 14 fungi all belonged to the Class Agaricomycetes, seven from Hymenochaetales, seven from Polyporaceae (Figure 9A). We also counted the homologous genes of *I. hispidus* and 14 other fungi species (Table 4). *I. hispidus* has 2,730 single-copy homologous genes and 1,323 multi-copy homologous genes in the species-shared gene family (Figure 9B). The gene family function enrichment analysis of *I. hispidus* and other 14 fungi showed that the top three enriched gene families of its unique GO annotated genes are “thiol-dependent deubiquitinase,” “cysteine-type endopeptidase activity,” and “ubiquitin-dependent protein catabolic process” (Figure 10A). A comparison of the KEGG database showed that the highest enriched gene families of *I. hispidus* are: “FoxO signaling pathway” (Figure 10B).

**TABLE 4:** Statistics table of gene family clustering results.

Species	Genes number	Genes in families	Family number	Unique families	Average genes per family
*P. noxium*	9930	9930	7737	1074	1.28
*F. mediterranea*	11330	11330	8129	1209	1.39
*S. baumii*	8455	8455	6606	359	1.28
*I. hispidu*	11845	11845	8352	1227	1.42
*P. nigrolimitatus*	14792	14792	9075	1775	1.63
*P. pouzarii*	8701	8701	6644	722	1.31
*S. paradoxa*	17088	17088	10514	3268	1.63
*R. mellea*	17134	17134	10045	2862	1.71
*W. cocos*	12213	12213	8464	1797	1.44
*G. sinense*	15478	15478	9494	1860	1.63
*P. arcularius*	17516	17516	11205	2714	1.56
*F. fomentarius*	14754	14754	10155	2033	1.45
*T. cingulata*	14108	14108	10572	2449	1.33
*T. coccinea*	12692	12692	9785	1682	1.3
*T. cinnabarina*	10229	10229	7611	314	1.34

**FIGURE 11:**
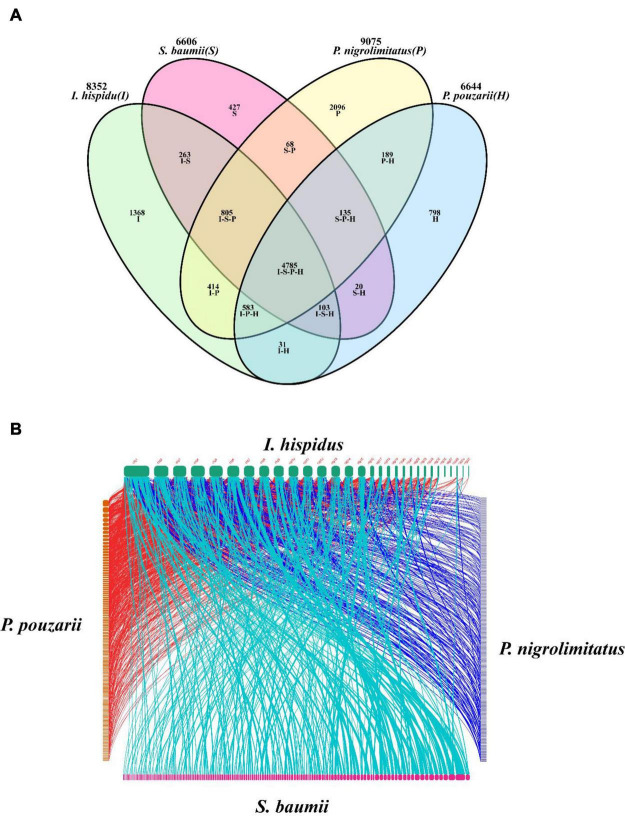
Comparative genomic analysis of *I. hispidus* and other three fungi. **(A)** Venn diagram of *I. hispidus*, *S. baumii, P. nigrolimitatus*, and *P. pouzarii*. **(B)** Genome synteny of *I. hispidus, S. baumii*, *P. nigrolimitatus*, and *P. pouzarii*.

**FIGURE 12:**
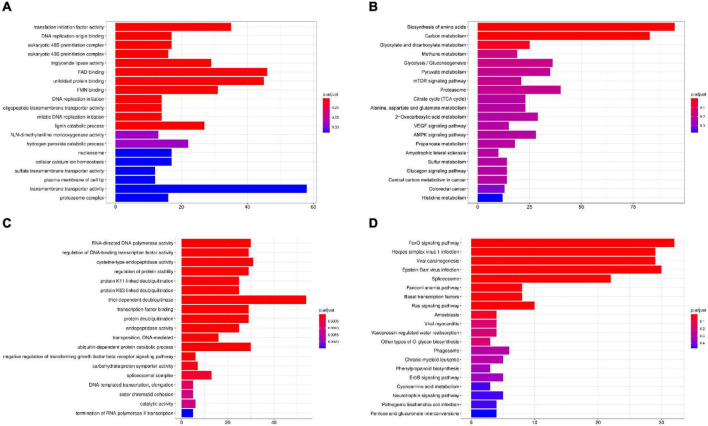
Gene family enrichment analysis of *I. hispidus* compared with *S. baumii, P. nigrolimitatus* and *P. pouzarii*. **(A)** GO enrichment results of the share gene family. **(B)** KEGG enrichment results of the shared gene family. **(C)** GO enrichment results of the unique gene family. **(D)** KEGG enrichment results of the unique gene family.

The results showed the highest homology among *I. hispidus, S. baumii, P. nigrolimitatus* and *P. pouzarii*. Therefore, we further performed the genome synteny analysis of these four fungi (Figures 11A,B). The gene families shared by these four fungi were subjected to GO database functional annotation analysis; the results showed that the four categories with the highest enrichment of genes shared by the four fungi were: “transmembrane transporter activity,” “FAD binding,” “unfolded protein binding,” and “translation initiation factor activity” (Figure 12A). The gene families shared by these four fungi were functionally annotated by the KEGG Pathway database; the results showed that the two categories with the highest enrichment of genes shared by the four fungi were: “biosynthesis of amino acids” and “carbon metabolism” (Figure 12B). Compared with the other three fungal *I. hispidus* unique gene families, GO functional annotation showed that the highest enrichment categories were “thiol-dependent deubiquitinase” (Figure 12C). Compared with the other three fungal *I. hispidus* unique gene families, KEGG Pathway functional annotation showed that the highest enrichment categories were: “FoxO signaling pathway” (Figure 12D).

## Discussion

*I. hispidus* is one of the key commercial products in agricultural production. However, few studies have investigated the genome sequence and genetic structure of *I. hispidus*. The genetic information of fungi can provide agricultural industry with data on the molecular mechanisms of host function and interactions with plants (Prasad Singh et al., 2019). Therefore, in this study, the whole-genome map of the *I. hispidus* was developed to explore the expression of its genetic information and the annotation of related functional genes. We obtained 999 protein sequences by cytochrome P450 annotation. Cytochrome P450 enzymes are heme thiol proteins that have been widely demonstrated to be involved in primary and secondary metabolism in fungi (Crešnar and Petrič, 2011). P450 enzymes can also participate in the detoxification process of exogenous substances, such as CYP504A1 and CYP504B1, and can degrade the benzene ring and its hydrocarbon derivatives (Ferrer-Sevillano and Fernández-Cañón, 2007). Furthermore, the P450 enzyme system can use benzoate as a substrate and exhibit O-demethylation activity, which is particularly important for the detoxification process of antifungal substances (Podobnik et al., 2008). The annotation results of cytochrome P450 are helpful for the subsequent development of functional genes of *I. hispidus*. Through the analysis of CAZymes, we found that the glycoside hydrolase family genes were significantly enriched in *I. hispidus*. The study conducted by Tao et al. found that glycoside hydrolase-related genes play a key role in the growth and development of mushrooms, and they are involved in the expansion of the cap (Tao et al., 2013). The results of another study showed that glycoside hydrolase-related genes can be significantly involved in the growth and senescence of edible mushrooms (Ding et al., 2007). Most glycoside hydrolase family genes also have the function of starch degradation, which means that the enrichment of these genes indicates that the *I. hispidus* contributes to nutrient absorption and utilization, and can use diverse substrates as energy sources (Fang et al., 2020). This evidence indicates that, at the gene level, the enrichment of glycoside hydrolase family genes contributes to the growth and the diversification of nutrient substrate utilization of *I. hispidus*. The yield of mushrooms depends on the utilization of substrates, and the degradation of lignocellulose is the most important part of the solution to substrate utilization (Ling and Fungi, 2018). Thus, it is important to study the composition of CAZyme in *I. hispidus* and determine its mechanism of action to provide a theoretical basis for the yield improvement of *I. hispidus* at the genetic level. Therefore, we annotated the CAZyme gene information based on the *I. hispidus* genome sequence. Our results can be used for the subsequent screening of candidate genes for the nutritional utilization of *I. hispidus* and the development of high-yielding and high-quality *I. hispidus* by genetic means.

This study has identified many essential genes related to secondary metabolites, which endow *I. hispidus* with diverse biological activities. Genes involved in the regulation of terpenes were most often found in the genome of *I. hispidus*. Triterpene structures in fungi play a crucial role in their biological functions. For example, triterpenes in *Ganoderma lucidum* (*G. lucidum*) can inhibit the proliferation of some cancer cells (Artursson et al., 2001). It showed significant toxicity to lung cancer and liver cancer cells. *In vitro* studies have shown that these triterpenoids inhibit the invasion and metastasis of cancer cells in vitro and may be used as a potential drug for cancer treatment (Chen et al., 2010). *G. lucidum* also showed a strong anti-tumor effect. In the experiment conducted by Gao et al., triterpenoids extracted from *G. lucidum* fruiting bodies inhibited tumor growth in mice with Lewis lung cancer (Gao et al., 2006). Moreover, terpenes showed vigorous antioxidant activity, significantly increasing the activities of superoxide dismutase and catalase, ultimately eliminating destructive reactive oxygen species (Ajith et al., 2009; Smina et al., 2011). *I. hispidus* has 35 genes involved in regulating terpenes, which explains the anti-tumor mechanism of this fungus at the genetic level. Further homology analysis demonstrated that *I. hispidus* and *S. baumii* showed high genetic similarity. The *S. baumii* extract also showed excellent antioxidant, anti-inflammatory, and anti-tumor activities (Wang et al., 2020; Sun et al., 2021; Zheng et al., 2021). In particular, *S. baumii* extract significantly induced a potential mitochondrial membrane breakdown and mitochondrial-dependent apoptosis in A375 cells (Wang et al., 2020). This also suggests that these similar genomes have the same biological activity.

By comparison of gene family enrichment analysis, we found that *I. hispidus*, *S. baumii*, *P. nigrolimitatus* and *P. pouzarii* shared the main category of “transmembrane transporter activity,” which performs the function of transporting substances from one side to the other. The transmembrane transporter family is associated with fungal growth and development (Saier, 1999). In addition to the common genes that function in the daily growth of fungi, compared with *S. baumii, P. nigrolimitatus* and *P. pouzarii*, the unique genes of *I. hispidus* are enriched in “thiol-dependent deubiquitinase” and “FoxO signaling pathway.” The FoxO signaling pathway involves many cellular physiological events such as apoptosis, cell cycle control, glucose metabolism, anti-oxidative stress and longevity (Flachsbart et al., 2009). Accumulated studies have found that FOXO can remove excessive free radicals and prevent oxidative damage. FOXO activates the expression of antioxidant enzyme systems (including promoting the expression of SOD and CAT) so that free radicals can be more effectively removed (Anselmi et al., 2009; Dansen et al., 2009). These may also be why I. hispidus has a strong antioxidant capacity.

In summary, in this study, the whole-genome map of *I. hispidus*, a crucial medicinal fungus, was developed for the first time. The Illumina NovaSeq high-throughput sequencing platform was used for sequencing. After establishing the database, the flow cell was transferred to the Oxford Nanopore PromethION sequencing instrument for real-time single-molecule sequencing. Functional annotations of the *I. hispidus* genome were performed using public databases and proprietary databases. Finally, the genes related to secondary metabolites were predicted; it was found that most of the genes involved in terpenes in the *I. hispidus* genome provided a theoretical basis for its medicinal value.

## Data availability statement

The datasets presented in this study can be found in online repositories. The names of the repository/repositories and accession number(s) can be found below: https://www.ncbi.nlm.nih.gov/, PRJNA848015 and https://www.ncbi.nlm.nih.gov/, SAMN28983551.

## Author contributions

ST performed the study and conducted data analysis. JX designed the research. LJ, PL, CS, SW, YY, YH, and RR provided assistance for the study. All authors read and revised the manuscript.

## Footnotes

1
https://fungismash.secondarymetabolites.org/#!/start
